# The development status of entrustable professional activities-based curricula in orthopedic and neurosurgical spinal surgery: a systematic review

**DOI:** 10.3389/fmed.2025.1575456

**Published:** 2025-12-05

**Authors:** Maximilian Domann, Fabiana Phillips, Martin R. Fischer, Matthias Stadler

**Affiliations:** 1 Institute of Medical Education, LMU University Hospital, LMU Munich, Munich, Germany; 2 Brainlab AG, Feldkirchen, Germany

**Keywords:** entrustable professional activities (EPAs), spinal surgery, medical education, postgraduate training, competency-based curriculum, systematic review, medical curriculum development

## Abstract

**Background:**

Postgraduate medical training often lacks clearly defined, assessable outcomes. Entrustable professional activities (EPAs) have emerged as a promising framework for competency-based training, aiming to improve transparency, accountability, and efficiency in specialist education. This systematic review investigates the current development status of EPA-based curricula in orthopedic and neurosurgical spinal surgery and highlights their potential to address educational challenges in this high-stakes field.

**Methods:**

A systematic review was conducted combining database studies (PubMed and Embase) and gray literature screening through professional societies’ websites and direct inquiries. Two reviewers independently screened, extracted, and synthesized the data. Given the anticipated heterogeneity among the included studies, a narrative synthesis was performed, while a quantitative synthesis was not feasible. No formal risk-of-bias assessment was conducted; however, potential sources of bias related to language, scope, and publication period were acknowledged and discussed.

**Results:**

Out of 15,768 initial records, only 4 relevant papers were identified. The EPAs in these studies were developed primarily through expert consensus methods such as modified Delphi or nominal group techniques. Additionally, among the 10 professional societies analyzed, only the AO Foundation (AO Spine) and the Royal College of Physicians and Surgeons of Canada provided explicit EPA frameworks for spinal surgery, contributing together to 26 EPAs. The limited number and heterogeneity of sources underscore the exploratory nature of this review and the current lack of standardized, spine-specific EPA curricula.

**Conclusion:**

EPA-based curricula in spinal surgery remain largely undeveloped. While conceptual frameworks exist, their implementation into structured residency programs is still in its infancy. This review provides a foundational overview of the existing EPAs and methodological approaches, offering a reference point for future curriculum development. Collaborative initiatives among professional societies are strongly encouraged to define, validate, and implement specialty-specific EPAs for spinal surgery, fostering competency-based education and improving training outcomes.

## 1 Background

The quality of postgraduate training for medical professionals is a key factor in the effectiveness of the healthcare system. A significant pillar of this training is known as “workplace learning,” which comprises practical work as a resident, theoretical work to further education courses, and self-study (1). Various “enablers” and “challengers” that either assist or hinder the learning process have been identified, often acting as bottlenecks (1). It was highlighted that active involvement in tasks is a positive aspect, but there is often an inappropriate level of responsibility placed on the learner, either too much or too little (1). Unclearly defined training goals or a lack of these goals also pose a substantial hurdle to the learning success of prospective doctors (1).

However, from the perspective of medical education research, these aspects can be influenced by an adequately structured specialist’s curriculum or appropriate teaching methods. In this study, the concept of “Entrustable Professional Activities,” or EPAs for short, is potentially highly valuable. More recently, those responsible for teaching in medical education have been giving growing attention to them, which represent a didactic concept that aims to create a competency-based training curriculum with a focus on acquiring specific, pre-defined competencies resulting in a more flexible training duration (2). The major characteristic of EPAs is the gradual decrease in the level of supervision until learners can eventually perform tasks entirely independently. This occurs at five levels, starting with level 1, which allows the learner to be present and observe, up to level 4, which attests to complete independence for the first time. Finally, level 5 additionally permits guiding younger learners independently (2, 3). It is crucial to emphasize that EPAs should not merely be viewed as learning objectives, as they represent specific professional activities (4). Thus, the entirety of EPAs, each representing a multitude of professional activities, collectively defines a profession, making a direct contribution to the healthcare system (2). By focusing a corresponding teaching concept primarily on acquiring competencies, it could contribute to shortening the training time for faster learners without reducing the quality of trained doctors (5). In general, substantial savings have already been reported when considering the entire medical education (6). Such a system would not only benefit medical students but also teaching staff and other employees and colleagues. They could clearly see which tasks may be performed with what degree of autonomy, thereby defining responsibilities clearly while simultaneously creating the possibility of evaluability (2, 4). In Europe, this concept received particular attention at the Charité in Berlin, where a study was conducted, ultimately identifying 12 central EPAs in 7 distinct categories (3).

However, when searching for Entrustable Professional Activities (EPAs) specific to specialist surgical training areas, it is observed that the majority of publications predominantly focus on EPAs in the domain of general surgery (7). Other surgical specialties or subspecialties, which include neurosurgical or orthopedic spinal surgery, have not yet been defined. This is despite the significant risk of these procedures, such as irreversible neurological damage, highlighting the need for an optimal training curriculum (8). Considering the medical-economic pressure, the professional training challenge of spinal surgery, and the possibility of promoting transparency and autonomy for aspiring doctors through EPAs, this study investigates the following research question: What is the current development status of curricula for orthopedic and neurosurgical spinal surgery based on EPAs?

## 2 Methods

To answer the research question, a systematic review was conducted, which included database research as well as a targeted gray literature strategy via professional societies, involving website searches and direct email outreach. Two reviewers screened titles and abstracts independently, as well as full texts, and two reviewers extracted data independently. Disagreements were resolved through discussion and, when necessary, with the help of a third reviewer.

Given the anticipated heterogeneity among the included studies, a narrative synthesis was planned, and a quantitative synthesis was not performed, as substantial methodological and outcome heterogeneity would obscure key comparative aspects.

No formal risk-of-bias assessment was conducted; however, potential biases related to language restriction, scope, and publication period were acknowledged and discussed within the limitations. Nevertheless, study design, context, and methodological transparency were narratively appraised to support interpretation. This review was not prospectively registered in PROSPERO or any other review registry.

### 2.1 Literature research

For the purpose of a thorough search of the existing literature on EPAs in orthopedic and neurosurgical spinal surgery, the PubMed and Embase databases were used. The following search terms were used in PubMed: “(EPAs) AND (neuro surgery),” “(Entrustable professional activities) AND (neuro surgery),” “(EPAs) AND (spine surgery),” “(Entrustable professional activities) AND (spine surgery),” “(EPAs) AND (orthopedic surgery),” and “(Entrustable professional activities) AND (orthopedic surgery)”; additionally, articles from the year 2005 onward were filtered because of their initial conceptualization in that same year. Duplicates were accordingly removed before evaluation, considering the following inclusion criteria: English or German languages, explicit mention of EPAs, and direct reference to spinal surgery from an orthopedic or neurosurgical perspective. At the same time, the following exclusion criteria were applied: Articles that discuss the necessity of Entrustable Professional Activities (EPAs) without providing specific recommendations for EPAs. This research took place from September 2023 to October 2025.

### 2.2 Medical professional societies

To increase the amount of literature about this topic, the websites of various professional societies, including their publications and direct statements obtained via email from these societies, were also included. The following professional societies were considered: German Society for Orthopedics and Orthopedic Surgery (DGOOC), German Society for Neurosurgery (DGNC), Swiss Institute for Medical Further and Continuing Education (SIWF), Swiss Society for Orthopedics and Traumatology (SGOT), American Board of Orthopedic Surgery (ABOS), American Board of Neurological Surgery (ABNS), North American Spine Society (NASS), American Association of Neurological Surgeons (AANS), Royal College of Physicians and Surgeons of Canada and the “Arbeitsgemeinschaft für Osteosynthesefragen (AO) Foundation, AO Spine.” In selecting the professional societies, an effort was made to gain a broad perspective by encompassing various geographical locations, reflecting both orthopedic and neurosurgical aspects of spinal surgery, and illustrating specific medical didactics. Furthermore, all institutions are representative in their fields of expertise and are among the largest and most relevant.

**Figure 1:**
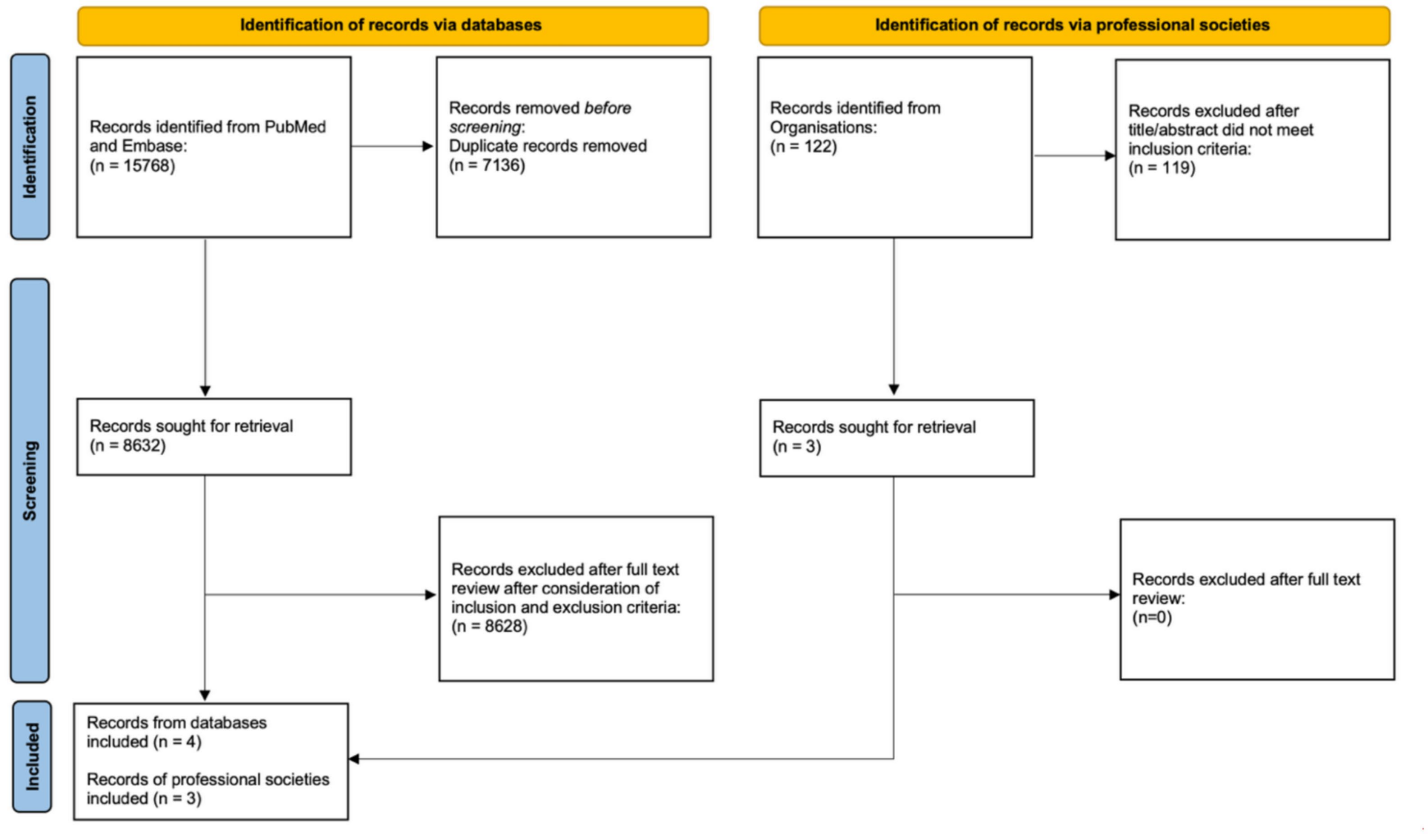
PRISMA 2020 flowchart of the publication screening and selection process.

For the publication-database search of professional societies, the term “Entrustable professional activities” was used from 2005 onward. As some websites did not allow filtering for publications only, all search results were included. This research took place from September 2023 to October 2025. Figure 1 visualizes both research approaches.

## 3 Results

### 3.1 PubMed search

After searching with the above terms, a total of 15,768 papers were found, with the number decreasing to 8,632 after the removal of duplicates. Ultimately, only four papers were included.

One of the papers developed EPAs based on existing orthopedic competencies, ultimately presenting a list of 49 EPAs for orthopedics, of which 4 were related to the spine, using a modified Delphi methodology (9). This paper included the following EPAs:

- Management of the patient with spinal cord injury
- Management of the patient with cauda equina injury
- Management of the patient with a lumbar herniated disc
- Management of the patient with spinal infection (9)

The second paper developed a competency-based European curriculum for neurosurgery through a competency-based, consensus-driven process following the European Union of Medical Specialists standards, involving expert consultation and iterative revisions within European neurosurgical education bodies. In total, five EPAs were embedded in the curriculum, of which one explicitly mentions spinal surgery and a second one may include spinal surgery as a “special interest training” (10). The following EPAs were listed:

- Emergency on-call neurosurgery (including the performance of cranial, spinal, peripheral nerves [where available], adult and pediatric neurological surgery)
- Elective operative neurosurgery (including advanced technical skills in one or two special interest areas) (10).

In the third paper, EPAs were developed through an expert consensus process led by the Training and Education Committee of the Korean Neurosurgical Society, incorporating input from various subspecialty societies to define. The program defines the final competencies expected of a neurosurgeon and specifies the corresponding core competencies and EPAs to achieve them. Its structure comprises 7 final competencies, 4 core competencies, 10 EPAs, and 12 key neurosurgical procedures, forming the foundation of a competency-oriented training framework (11).

In this program, several of the EPAs formulated reflect the general management of neurosurgical patients; however, only the “EPA 4: neurosurgical procedures available: decompressed laminoplasty” is directly related to the EPAs relevant to spine surgery (11).

In the fourth paper, relevant EPAs were identified using a multistep nominal group technique involving experienced neurovascular specialists and a medical educator, with iterative feedback rounds until consensus was achieved. Although this study focuses on vascular neurosurgery, one of the formulated EPAs, “Spinal Dural Arteriovenous Fistula,” is included here due to its direct anatomical relevance to the spine. The remaining EPAs, however, primarily cover general preoperative and postoperative management as well as other surgical skills not directly related to the spine (12).

Finally, it is noteworthy that the EPA selection from the AO Spine Curriculum (13) was made explicitly due to its direct relevance to the surgical aspect of spinal surgery. Nevertheless, additional EPAs were listed that should not be viewed as either “exhaustive” or “exclusive” according to the curriculum, but rather as examples illustrating spinal surgery (13). For this reason, some EPAs are mentioned that do not directly contribute to surgical training but also exemplarily represent other aspects of teaching, quality management, or the billing system (13). The more general nature of the EPAs, which belong to the holistic profession, was the reason for excluding these and focusing on the operative ones. It is also worth noting that the so-called Core-EPAs, upon which the curriculum is based, are also not mentioned due to their general nature (13). The selected EPAs are as follows:

- Assessment of and consent from the preoperative patient
- Position the patient
- Perform a preoperative team briefing
- Perform the procedure safely
- Document the procedure
- Transfer the patient to the recovery area
- Check the patient postoperatively (13)

### 3.2 Results of the professional societies search

Table 1 summarizes all results of the professional societies search in detail. Notably, out of a total of 10 professional societies, only 2 had specific publications in their database that address EPAs in spinal surgery. Only three other societies yielded results related to EPAs, but none of them were related to spinal surgery. Of these three, the SIWF featured a dedicated website tab for EPAs and, from 2005 onward, had the highest number of search results with 120 publications; however, none specifically addressed spine surgery. Only the Royal College of Physicians and Surgeons of Canada, as well as the AO Foundation, AO Spine, with their AO Spine Curriculum Second Edition, defined EPAs for spinal surgery, totaling 26 (13–15). Notably, the Royal College of Physicians and Surgeons of Canada provides an extensive selection of EPAs across multiple fields of specialization, contributing 19 of the 26 EPAs identified. Here, both neurosurgical EPAs related to the spine and orthopedic EPAs were identified, resulting in duplication, as the EPA “Applying external spinal fixation and/or traction” appears in both the orthopedic and neurosurgical contexts. Table 1 summarizes all EPAs that have been found during this research process. Furthermore, Table 2 provides an overall summary of all studies and documents that have been identified.

**Table 1:** List of professional medical societies and their publications on entrustable professional activities (EPAs).

Professional society	Database research	Inquiry
AO Foundation, AO Spine	A total of two publications, one of which has a direct relation to EPAs in spinal surgery.	No additional inquiry necessary.
German Society for Orthopedics and Orthopedic Surgery (DGOOC)	A total of three search results, but none related to EPAs.	No response to our inquiry.
German Society for Neurosurgery (DGNC)	No publications.	There is no such catalog from the NCA to date.
Swiss Institute for Medical Further and Continuing Education (SIWF)	There is a total of 120 search results for the term “Entrustable Professional Activities,” none of which were related to spinal surgery EPAs.	Pending response from the spine subgroup.
Swiss Society for Orthopedics and Traumatology (SGOT)	No publications.	No preliminary work is in progress, let alone finalized. However, there is a planned implementation set for 2027.
American Board of Orthopedic Surgery (ABOS)	No publications.	No direct research, but partners with researchers.
American Board of Neurological Surgery (ABNS)	No publications.	No response to our inquiry.
North American Spine Society (NASS)	No publications.	There is no EPA-based training provided by NASS, and none is currently being developed.
American Association of Neurological Surgeons (AANS)	There was a total of three publications, but only two of them were related to the topic of EPAs. However, none of them were related to spinal surgery.	No response to our inquiry.
Royal College of Physicians and Surgeons of Canada	Multiple EPA guidelines for different fields of specialization are available.	No additional inquiry necessary.

**Table 2:** Summary of explicitly named entrustable professional activities (EPAs) from the PubMed search and the professional societies search.

Source	EPAs identified	Setting/Method
Watson et al. (2021) (9)	Management of the patient with spinal cord injury	Modified Delphi methodology.
	Management of the patient with cauda equina injury.	
	Management of the patient with a lumbar herniated disk.	
	Management of the patient with spinal infection.	
Whitfield et al. (2023) (10)	Emergency on-call neurosurgery (including crania, spinal, peripheral nerve, adult, and pediatric neurological surgery).	Competency-based, consensus-driven process following the European Union of Medical Specialists standards, involving expert consultation and iterative revisions within European neurosurgical education bodies.
	Elective operative neurosurgery (including advanced technical skills in special interest areas).	
Park et al. (2025) (11)	EPA 4: neurosurgical procedures available: decompressed laminoplasty.	Expert consensus process led by the Training and Education Committee of the Korean Neurosurgical Society, incorporating input from various subspecialty societies to define.
Van Lieshout et al. (2023) (12)	Spinal dural arteriovenous fistula.	Multistep nominal group technique involving experienced neurovascular specialists and a medical educator, with iterative feedback rounds until consensus was reached.
AO Spine Curriculum (13)	Assessment of and consent from the preoperative patient.	Consensus process using the Delphi method.
	Position the patient.	
	Perform a preoperative team briefing.	
	Perform the procedure safely.	
	Document the procedure	
	Transfer the patient to the recovery area.	
	Check the patient postoperatively.	
Royal College of Physicians and Surgeons of Canada – Orthopedic EPAs (15)	Applying external spinal fixation and/or traction.	Royal College Specialty Committee–led expert consensus under the Competence by Design framework; national stakeholder consultation and iterative revisions.
	Performing primary posterior instrumented spine fusions.	
	Performing laminectomy/decompression.	
	Performing posterior spinal column exposure and closure	
Royal College of Physicians and Surgeons of Canada – Neurosurgery EPAs (14)	Providing initial management for patients with a spinal emergency.	Royal College Specialty Committee–led expert consensus under the Competence by Design framework; national stakeholder consultation and iterative revisions.
	Applying external spinal fixation and/or traction.	
	Performing midline posterior subaxial spinal column exposure and closure.	
	Providing neurosurgical consultation for patients with non-urgent spinal conditions.	
	Providing definitive management for patients with spinal emergencies.	
	Performing lumbar laminectomy (JC).	
	Exposing the anterior cervical spine (JC).	
	Performing lumbar microdiscectomy (SC).	
	Performing posterior cervical or thoracic decompression (SC).	
	Performing anterior cervical decompression (SC).	
	Performing procedures utilizing spinal instrumentation, including posterior subaxial, posterior thoraco-lumbar, occipito-cervical, and anterior cervical (SC).	
	Providing surgical management of spinal intradural lesions (SC).	
	Neurosurgical consultation for patients with non-urgent cranial and spinal vascular conditions.	
	Providing neurosurgical consultation for patients with urgent cranial and spinal vascular conditions.	
	Performing spine procedures for pediatric patients.	

## 4 Discussion

This study explored the current developmental status of EPA-based curricula in orthopedic and neurosurgical spinal surgery. This inquiry is motivated by lengthy and costly training periods in medical education, which could potentially be shortened through the innovative concept of EPAs. To investigate this, a systematic review was conducted, including searches of the PubMed and Embase databases and contact with numerous professional societies.

The results, more specifically the lack of results, of the exploratory question indicate that while the “EPAs” concept is highly popular, it has only been sporadically integrated into further education. In particular, at the beginning of medical residency, more tangible information can be found. Despite the large number of initial results, only four ultimately relevant papers underscore the topic’s popularity while also reflecting the limited specificity of existing studies. The paper sets their EPAs as benchmarks for future curriculum designs, indicating that actual implementation into the daily training routine of doctors has only been carried out in terms of theoretical preparatory work or is in a very early stage of implementation. In line with this finding, it is important to note that these frameworks remain largely conceptual, with little documented evidence of implementation or measurable educational outcomes to date. This highlights that the identified EPA structures reflect a developmental or aspirational stage rather than an established educational practice.

A comparable picture is provided by the professional societies. Several societies appear interested in EPAs, but only the “AO Foundation, AO Spine” and the “Royal College of Physicians and Surgeons of Canada” provide actual work on spine surgery EPAs. The remaining societies either have not provided current work on EPA-based curricula, or no evidence was found in their databases. This further underscores the lack of publications on this topic, thus underlining the relevance of the research question and clearly demonstrating that EPA-based curricula in spinal surgery represent a gap in research. However, the limited number of sources constrains the generalizability of our findings. As such, this review primarily reports the existence of a curricular gap rather than assessing the quality or effectiveness of proposed EPA-based training structures.

This limited integration of EPA-based curricula in spinal surgery may, in part, be explained by the inherent complexity and resource demands of EPA implementation. While EPAs offer a promising extension of competency-based training (16), their development requires both the translation of medical knowledge into observable, measurable competencies and significant personnel investment—particularly in the early, supervision-intensive stages of training (2). In highly specialized fields such as spinal surgery, where precision and safety are paramount, institutions may be hesitant to adopt novel frameworks without robust implementation strategies. Furthermore, the flexibility EPAs introduce—allowing for shortened or extended training durations—may pose logistical and financial challenges in structured hospital environments (2). Compared to broader fields such as general surgery, which have already begun to incorporate EPAs more widely, subspecialties such as spine surgery may lag behind because of slower curriculum reform and the added complexity of defining and validating specialty-specific EPAs (2).

To bridge this gap, we recommend using the results of this study as a starting point to have an initial collection of current EPAs for spinal surgery. To specify more EPAs, the approach of Watson et al. (2021) would be suitable to derive an expert consensus on spinal EPAs from a preliminary EPA list using their modified Delphi methodology, ensuring a comprehensive representation of spinal surgery. Furthermore, we specifically recommend using Ten Cate’s eight sections for a complete EPA description (2), as describing them through so-called “nested EPAs”—that is, EPAs within an overarching EPA—appears particularly useful in the context of spinal surgery. Finally, research and development among professional societies should be encouraged. Especially the current EPAs of the “AO Foundation, AO Spine” and the “Royal College of Physicians and Surgeons of Canada” could be used to start a collaborative effort to further or even newly develop EPAs.

In interpreting the results of this systematic review, it is important to keep in mind that only German and English literature was included. However, this decision was made because the first competency-based postgraduate medical training programs originated from Canada’s CanMEDS competencies and the ACGME in the USA (17, 18). Additionally, Switzerland is a pioneer, as it introduced a competency-based catalog in 2017, called PROFILES, at least for medical education, which includes not only the CanMEDS framework but also specific EPAs (19, 20). Nevertheless, publications in other languages, such as French, Spanish, or Chinese, could provide valuable insights into further EPAs, which is why future research in this regard should be undertaken. The narrow focus on spinal surgery also leads to fewer findings; however, explicitly due to the high relevance of this field and its potentially fatal consequences in case of errors, the search was conducted so strictly and closely. Finally, filtering from 2005 onward is also a limitation, but it was chosen due to the introduction of the specific didactic methodology of EPAs by Cate in the same year (16). Overall, a practically unexplored field of EPAs for spinal surgery is emerging, whereas this situation could also apply to other specializations, which should be the subject of future research. It will be necessary not only to advance the implementation of general EPA-based curricula but also to become more specific, particularly in the context of specialist medical training.

## 5 Conclusion

This systematic review has shown that the literature on EPA-based curricula in spinal surgery is scarce, and professional societies are equally sporadic in their current work on corresponding educational concepts. In the context of the promising potential of EPAs and the time-consuming, costly nature of medical training, further research and implementation efforts are warranted. Together with the results and the suggested potential EPAs for this discipline, this systematic review offers a benchmark for future studies and aims to encourage continued research in the field of spinal surgery.

## Data Availability

The original contributions presented in the study are included in the article/supplementary material, further inquiries can be directed to the corresponding author.
